# Modification of hemp shiv properties using water-repellent sol–gel coatings

**DOI:** 10.1007/s10971-018-4621-2

**Published:** 2018-03-10

**Authors:** Atif Hussain, Juliana Calabria-Holley, Yunhong Jiang, Mike Lawrence

**Affiliations:** BRE Centre for Innovative Construction Materials, Department of Architecture and Civil Engineering, University of Bath, Bath, BA2 7AY UK

**Keywords:** Sol–gel, Dip-coating, Water repellence, Hygroscopic, Bio-based materials

## Abstract

For the first time, the hydrophilicity of hemp shiv was modified without the compromise of its hygroscopic properties. This research focused on the use of sol–gel method in preparation of coatings on the natural plant material, hemp shiv, that has growing potential in the construction industry as a thermal insulator. The sol–gel coatings were produced by cohydrolysis and polycondensation of tetraethyl orthosilicate (TEOS) using an acidic catalyst. Methyltriethoxysilane (MTES) was added as the hydrophobic precursor to provide water resistance to the bio-based material. Scanning electron microscopy (SEM) and focused ion beam (FIB) have been used to determine the morphological changes on the surface as well as within the hemp shiv. It was found that the sol–gel coatings caused a reduction in water uptake but did not strongly influence the moisture sorption behaviour of hemp shiv. Fourier transformed infrared (FTIR) spectroscopy shows that the coating layer on hemp shiv acts a shield, thereby lowering peak intensity in the wavelength range 1200–1800 cm^−1^. The sol–gel coating affected pore size distribution and cumulative pore volume of the shiv resulting in tailored porosity. The overall porosity of shiv decreased with a refinement in diameter of the larger pores. Thermal analysis was performed using TGA and stability of coated and uncoated hemp shiv have been evaluated. Hemp shiv modified with sol–gel coating can potentially develop sustainable heat insulating composites with better hygrothermal properties.

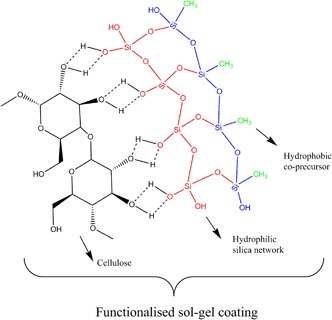

## Introduction

The use of bio-based materials (derived from plant sources) have become increasingly popular to produce economical engineering materials in the construction industry [1]. Bio-based materials have numerous advantages over conventional non-renewable building materials such as lower embodied energy, lower CO_2_ emissions of buildings and demand for in-use energy can be significantly reduced through passive environmental control [2]. Other advantages of bio-based materials include good specific strength, lower density, economic viability, biodegradability, non-irritant nature and good heat capacity [3].

Several studies have discussed the ability of bio-based materials used in construction to absorb and release moisture in response to changes in relative humidity (RH) in the surroundings which creates a breathable wall. These materials act as a hygric buffer and eventually reduce the energy demands for air conditioning [4]. The response to varying humidity conditions is linked to their pore structure and pore connectivity where the moisture condenses and evaporates on the surface of the material and within its pores. This leads to an increased effective thermal mass, allowing the bio-based material to behave as a thermal buffer in addition to their hygric buffering characteristics [5].

Towards the end of the 20th century, a bio-based building material was rediscovered which used the woody core or shiv of hemp (Cannabis Sativa L.) and lime based binders to produce hemp concrete. Hemp shiv has very low conductivity compared to lime due to its porous structure. Many studies have been conducted over the recent years to optimise the hemp characteristics, curing conditions and binder content during the production of hemp based concrete. Hemp insulation materials show good hygrothermal properties by regulating humidity inside buildings and has low environmental impact [6, 7]. The major constituents of industrial hemp shiv are: cellulose (44%), hemicellulose (18–27%), lignin (22–28%) and other components such as extractives (1–6%) and ash (1–2%) [8].

Considering bio-based insulation material to be a part of a vapour permeable wall, significant benefits can be achieved such as better indoor air quality [9] and robustness of fabric. For example when moisture is allowed to penetrate through the fabric of the wall the risk of moisture build-up is considerably reduced [10]. Under suitable environmental conditions, bio-based materials are durable and long-lasting. However, in presence of excess moisture, these materials are susceptible to decay and therefore it is not advisable to use them below damp proof courses or in areas which could get wet.

Hemp shiv has tendency to absorb large amounts of water due to its highly porous structure and presence of hydrophilic hydroxyl groups in its structure. This leads to certain disadvantages of using bio-based materials making them incompatible with hydrophobic thermoset/thermoplastic polymers causing poor adhesion in the matrix interface of the composites [11]. There is a high competition between the binders used with bio-based material due to the wide use of different binders in construction. Since the shiv competes with the binder for the available water, purely hydraulic binders like lime or cement cannot hydrate completely, leading to a powdery inner core in the hemp-lime walls which is poorly bound.

As a result, during the manufacture of hemp concrete, water is added in significant excess amounts compared to what is actually needed for the hydration of lime. This leads to long drying times ranging from several months to over a year which are not acceptable to be employed at an industrial scale [12]. Large water absorption capacity of bio-based materials can even cause problems in the end product stage when undesirable water comes in contact or if the surroundings are humid. Previous studies have reported that hemp shiv not only has higher water absorption rate but also absorb high amounts of water in the very first minutes compared with other plant materials [13].

Several studies have reported the improvement in mechanical properties of natural-fibre composites through alkali [14, 15], acetyl [16] and silane [17, 18] treatment of plant fibres. The chemical treatments react with the hydroxyl groups and improve the hydrophobic characteristics of fibres [19, 20]. The sol–gel technique is a highly versatile method to deposit silica based coatings possessing single or multi functionality [21–23]. These thin mesoporous coatings have high structural homogeneity and their adhesion can be tailored to different substrates [24, 25]. Sol–gel based hydrophobic and water-repellent coatings have been investigated on different plant based materials such as wood [26, 27] and cellulosic based materials [28–30]. Wood and cellulosic fibres modified with sol–gel material showed significant reduction in flammability and enhanced fire-resistance properties. [31, 32].

The objective of this work was to treat the hemp shiv with a silica sol–gel coating functionalised with a hydrophobic agent in order to foster hydrophobicity of the hemp shiv. Our work focuses on creating a breathable coating around the shiv and investigate how the change in surface chemistry can alter the physical properties of the hemp shiv.

## Materials and methods

Hemp shiv used in this study was received from CAVAC, an agricultural cooperative based in northwest France. The sol–gel was synthesised by hydrolysis and condensation of tetraethyl orthosilicate (TEOS) in ethanol and water. The reaction was catalysed by nitric acid. 1 M of TEOS was added to a mixture of 8 M distilled water, 4 M of absolute ethanol and 0.005 M of nitric acid. 0.33 M of methyltriethoxysilane (MTES) was added to the above mixture as the hydrophobic agent. Finally, 0.33 M of drying control chemical additive N,N-dimethylformamide was added. The sol was vigorously stirred at 40 °C and atmospheric pressure for nearly 2 h. All the chemicals were obtained from Sigma-Aldrich.

Gelation took place in situ in which pieces of hemp shiv were dipped in the sol for 10 min and then carefully removed and transferred onto a Petri dish. The samples were placed in an oven at 40 °C for 1 h and then dried at 80 °C for 2 h. The residual water content was calculated by sealing the oven dried samples in a glass tube under vacuum, heating them at 150°C overnight and then weighing the sample. The amount of residual water was 5 wt% for the sol–gel coated samples.

For preparation of the silica the sol was allowed to age in a container to a gel state at room temperature for 48 h The gel underwent dehydration at 80 °C for 120 h to obtain the silica.

### Surface morphology

Photomicrographs of coated and uncoated hemp shiv samples were captured using a dual beam focused ion beam (FIB) system model FEI Helios NanoLab 600. This system is equipped with an extremely high resolution Elstar scanning electron microscopy (SEM) column and a fine-probe ion source. A high beam current of gallium ions was used for site specific sputtering and milling to prepare a specific area within the sample. All the samples were gold coated using an Edwards Scancoat Gold Sputter Coater. A further layer of platinum was deposited on the areas where higher beam current of gallium ions was used.

### Water absorption test

To eliminate initial moisture content, the hemp shiv samples were dried overnight in an oven at 80 °C and then weighed to the nearest 0.1 mg. The samples were then completely immersed in water without using any external force. Since the density of shiv is lower than water, the samples were expected to float. Hence most of the water uptake observed was due to capillary action. The samples were removed at frequent intervals, shaking off any visible surface water and weighed to the nearest 0.1 mg within 30 s of removal from water. Mass readings were taken regularly for the next 24 h and the water absorption was calculated by the mass change (%). The readings reported were average of three measurements.

### Dynamic vapour sorption

Isotherm analysis of uncoated and coated hemp shiv was carried out using a dynamic vapour sorption apparatus (DVS Advantage, Surface Measurement Systems). Hemp shiv samples were prepared weighing ~15 mg and placed on the sample holder, combined with a microbalance by a hanging wire. The instrument was maintained at constant temperature of 23 °C and the RH was increased in steps in the following sequence (0, 10, 20, 30, 40, 50, 60, 70, 80 and 90% RH), before decreasing to 0% RH in the reverse order. Each RH step change was programmed to move to the next when the moisture content was stable for duration of at least 10 minutes (d*m*/d*t* < 0.002%). However, it should be noted that the maximum time allowed for each RH step to reach stability was 360 min. Previous studies have established that this value allows for obtaining equilibrium moisture content (EMC) values within 0.1% of the true equilibrium value [33]. The target RH, actual RH, running time and sample mass were recorded throughout the isotherm run.

### Calculation of moisture content

Moisture content was calculated using the DVS data based on the mass of treated and untreated shiv as per the following equations:

1 $${\rm {MC}} = \frac{{m_2 - m_1}}{{m_1}} \times 100$$

2 $${\rm {MC}}_{\rm {R}} = \frac{{m_2 - m_1}}{{m_0}} \times 100$$

where MC is the measured EMC of uncoated and coated shiv; MC_R_ is the reduced EMC of coated shiv based on the mass of shiv before coating; *m*_0_ is the dry mass of shiv before coating; *m*_1_ is the dry mass of shiv after coating; *m*_2_ is the equilibrium mass of shiv at a given RH.

From the above equations, it is clear that for the uncoated hemp shiv, EMC = MC = MC_R_. MC takes no account of the fact that the mass of the sample is increased due to the sol–gel coating layers. MC_R_, however, reflects the effect of deposition of sol–gel coating layers on the adsorption–desorption isotherms of hemp shiv. However, Eq. (2) is not realistic as we are assuming that the sol–gel coating does not adsorb any moisture. To differentiate between surface effect and pore volume effect, Eq. (1) was modified to employ volume instead of mass. The new equation would be:

3 $${\rm {MC}}_{\rm {vol}} = \frac{{v_2}}{{v_1}} \times 100$$

where MC_vol_ is the moisture content by volume of water adsorbed, *v*_1_ is the total accessible volume of the sample; *v*_2_ is the volume of adsorbed moisture at a given RH.

### Porosity

The pore size distribution test was performed by using Thermo Scientific Pascal Mercury Porosimeter Model 140 for low pressure and Model 440 for high pressure. The pressure range for the test was between 0.1 KPa to 400 MPa and the pore size measuring range was 116 μm to 3.6 nm. Pressure, pore diameter and intrusion volume were automatically registered.

### Fourier transform infrared spectroscopy

Fourier transform infrared (FTIR) analysis on treated and untreated hemp shiv was carried out by using a PerkinElmer FTIR spectrometer model Frontier. Transmittance spectra were collected with 2 cm^−1^ resolution and 10 scans were accumulated for each spectrum in the range 4000–600 cm^−1^. For analysis of the silica, specimens were crushed into powder and then grounded with KBr to produce pellets.

### Thermal analysis

Thermal analysis of the uncoated and sol–gel coated samples was conducted by thermogravimetric analysis (TGA) using equipment STA 449 F1 Jupiter (Netzsch, Germany). The samples were heated at a rate of 10 K min^−1^ from 25 to 800 °C under nitrogen atmosphere purged at 30 ml min^−1^ using an alumina crucible.

## Results and discussion

**Fig. 1:**
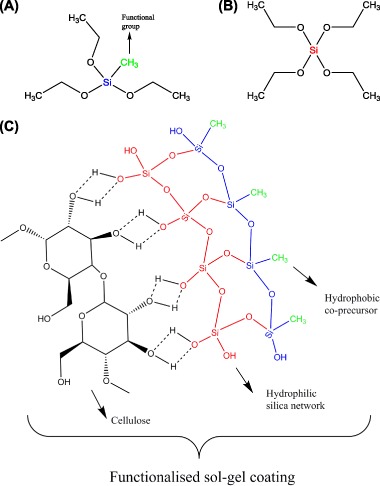
Structure of an **a** MTES molecule, **b** TEOS molecule and **c** scheme for deposition of sol–gel coating on shiv surface

The water-repellent sol–gel coatings were prepared using MTES as the additive during the sol synthesis. In the present work, the co-precursor method of sol–gel synthesis was followed based on the simplicity of the process. In the sol–gel process, TEOS is hydrolysed and condensed to form a SiO_2_ network which is linked to the raw material through the hydroxyl sites of cellulose present in the hemp shiv. On addition of MTES as a co-precursor during the sol–gel processing, the hydroxyl groups on the silica clusters are replaced by the –Si–CH_3_ groups through –O–Si–CH_3_ bonds as seen in Fig. 1. The hydrophobicity of the sol–gel coatings is due to the attachment of –Si–CH_3_ groups on the SiO_2_ network through oxygen bonds. Hence, by increasing the numbers of layers of the sol–gel coating a reduction of the hydroxyl sites on cellulose was obtained. Conversely the number of –SiCH_3_ groups increased, which provided increased performance of the substrate (shiv) against water repellence.

### Characterisation of the surface morphology

**Fig. 2:**
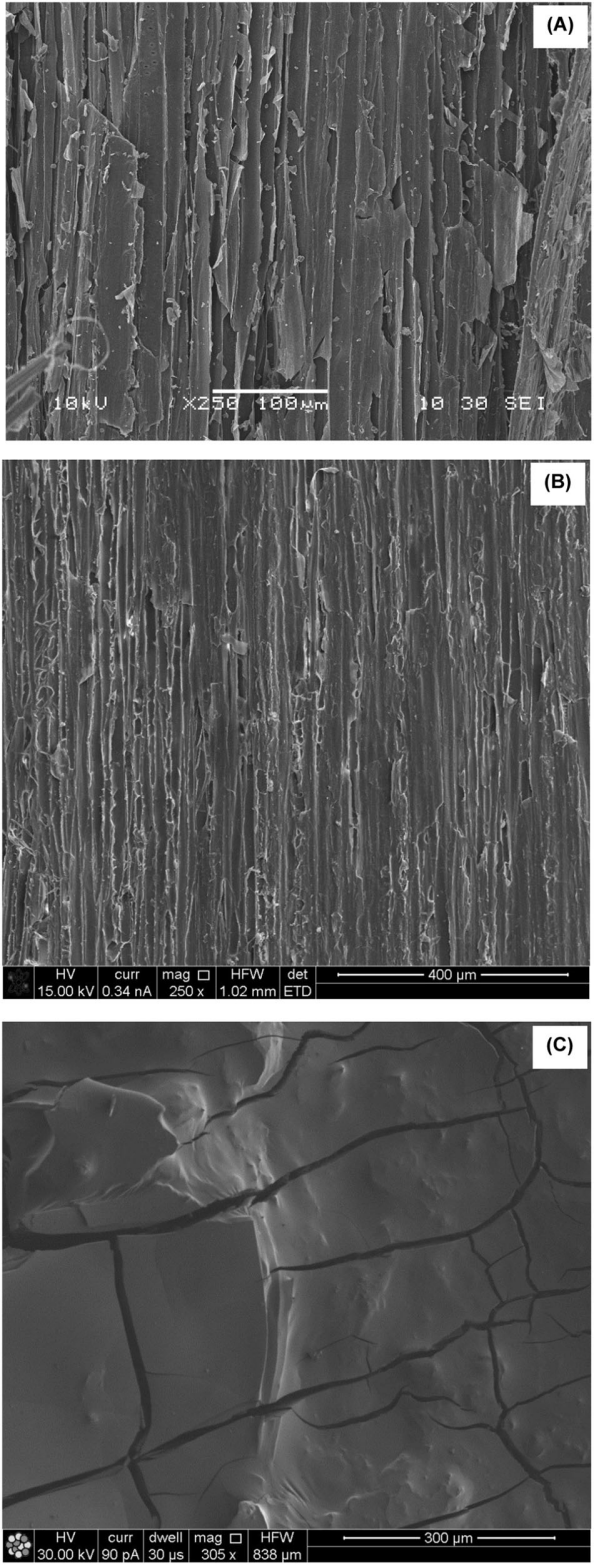
SEM micrographs of hemp shiv **a** uncoated, **b** one layer of sol–gel coating and **c** ten layers of sol–gel coating

The sol–gel coating affected the surface morphology of the hemp shiv. Figure 2 shows the SEM micrographs of the surface of hemp shiv before and after treatment. Hemp shiv treated with a single layer of sol–gel coating demonstrates a more uniform surface compared to the untreated shiv. However, when the hemp shiv was treated with ten layers of the sol–gel coating, the formed film showed extensive cracks as seen in Fig. 2c.

**Fig. 3:**
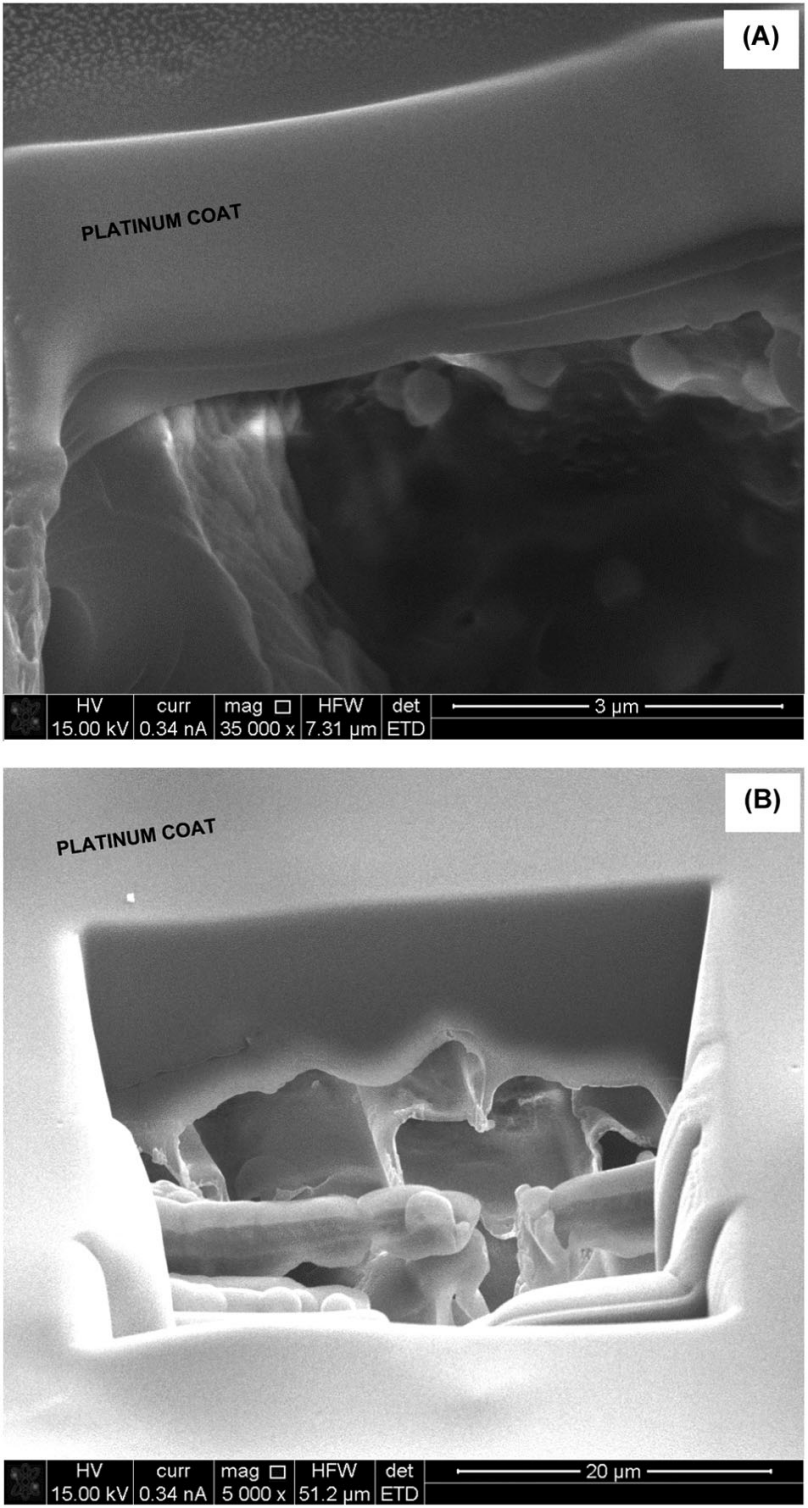
SEM/FIB micrographs of hemp shiv **a** one layer of sol–gel coating and **b** ten layers of sol–gel coating

The morphology of the sol–gel modified hemp shiv was determined from an FIB cross section. Figure 3 shows the SEM micrographs of the coating layer. FIB was used to cut a section (40×30 µm and around 20 µm deep) on the surface of hemp shiv. Thin cracks were observed in the sol–gel coating formed on the surface of shiv. Ten layers of the sol–gel coating provided complete shielding of the shiv surface. FIB cross section of this piece of shiv showed that the sol–gel coating had penetrated deep into the shiv.

**Fig. 4:**
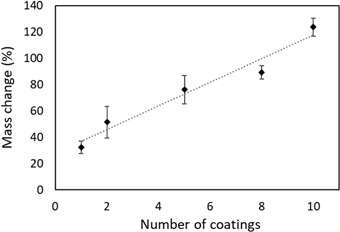
Mass gain of hemp shiv with sol–gel coatings

The sol–gel coating increased the mass of the hemp shiv as seen in Fig. 4. During the first dip, most of the sol is absorbed into the cell wall of the shiv resulting in a very thin film on the entire shiv surface including pits. A single layer of sol–gel coating on the shiv resulted in 30% overall mass gain. This is in agreement with sol–gel coatings on wood resulting in 25–35% mass gain due to the absorption of coatings within the cell wall [27, 34]. Further coatings on the same piece of shiv showed that the mass gain followed a linear trend. Further layers resulted mainly in an increased thickness of the coating on the shiv surface as well as within the pits thereby the effect was a shielded pore structure as seen with the SEM (Fig. 3b).

### Characterisation of surface chemistry

**Fig. 5:**
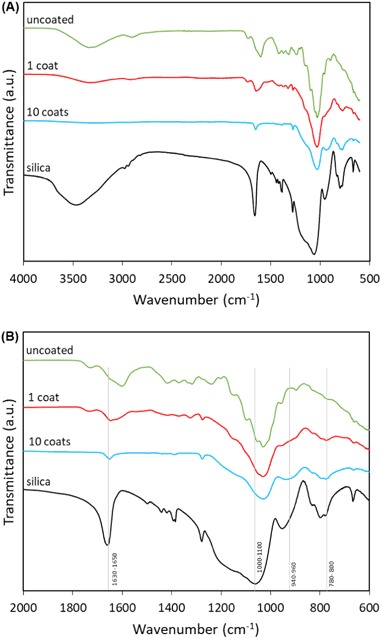
FTIR spectra of silica, uncoated and coated hemp shiv in **a** 600–4000 cm^−1^ region and **b** 600–2000 cm^−1^ region

Figure 5 shows the FTIR spectra of uncoated and sol–gel coated hemp shiv and the peaks are listed in Table 1. The reduction of free water bands corresponding to the wave number interval 3300–3400 cm^−1^ in the coated shiv indicates that the coating has enhanced the water repellence of hemp shiv. Other wavenumbers that confirm the presence of sol–gel coating on the surface of shiv are 940 cm^−1^ corresponding to vibration of Si–OH bonds and 780 cm^−1^ associated with molecules due to incomplete hydrolysis of TEOS. It is also observed in Fig. 5b that coating the shiv results in loss of peak intensity in the region 1200–1800 cm^−1^ thereby masking the functional groups present on the surface of hemp shiv.

**Table 1:** FTIR peaks corresponding to the source [15, 35–37]

Wavenumber (cm^−1^)	Vibration	Source
3341	O–H stretch	Polysaccharides
2918	C–H vibration	Polysaccharides
2851	CH_2_ stretch	Wax
1742–1733	C=O stretch in unconjugated ketone, carbonyl and ester groups	Hemicellulose, wax
1605–1639	C=C stretch	Lignin
1630–1660	−OH	Adsorbed water
1424	CH_2_ bending, C=C stretching in aromatic group, CH in plane deformation	Cellulose, lignin
1373	CH bending	Cellulose
1319	C–C stretch, CH_2_ vibration	Lignin, cellulose
1027	C–C, C–OH, C–H ring and side group vibration	Hemicellulose, pectin
1000–1100	Si–O–Si	Silica
940–960	Si–OH	Silica
896	C–O–C glycosidic stretch, O–H bending	Polysaccharides
780–800	Si–O–Si, SiOCH_2_CH_3_—incomplete hydrolysis of TEOS	Silica

The region below 1000 cm^−1^ highlight the presence of Si–OH and Si–O–Si bonds although this cannot prove the covalent grafting of sol–gel coating onto C–OH of cellulose or hemicellulose. From the FTIR signals, one can prove, at best, that silica has been added on to the hemp shiv surface. For confirmation of covalent bonding between hemp shiv and the sol–gel network, further analysis is recommended.

### Water absorption

The percentage of distilled water absorption (WA %) for hemp shiv was calculated using the following equation:

3 $${\mathrm{WA\% }} = \frac{{{\mathrm{Sample}}\,{\mathrm{wet}}\,{\mathrm{weight}} - {\mathrm{Sample}}\,{\mathrm{dry}}\,{\mathrm{weight}}}}{{{\mathrm{Sample}}\,{\mathrm{dry}}\,{\mathrm{weight}}}} \times 100$$

**Fig. 6:**
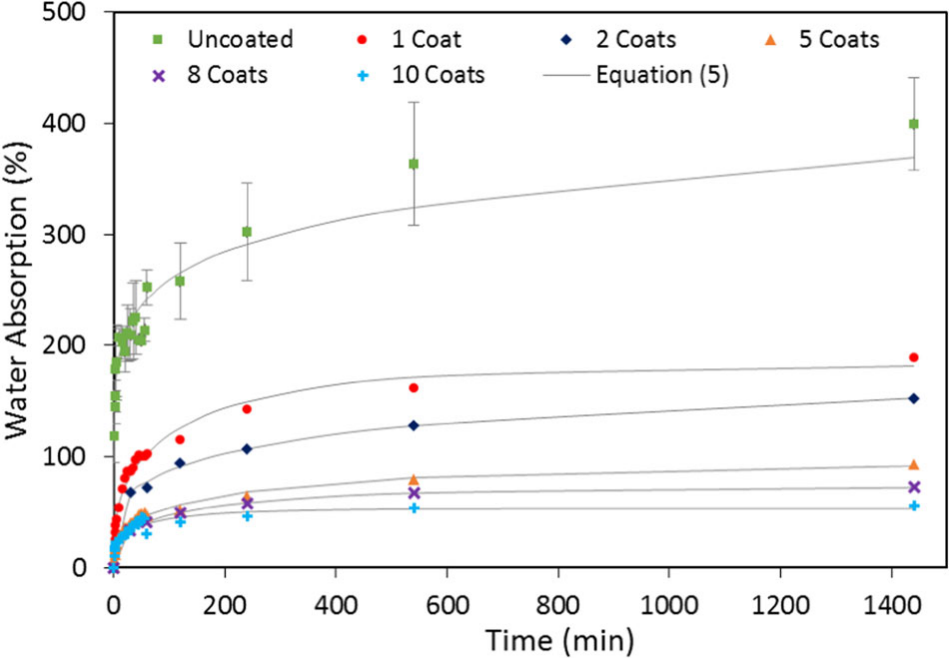
Dependence of water absorption on time for varying coating layers on hemp shiv

It is a measure for percent relative increase in weight due to water retention within the sample. Figure 6 depicts the distilled water absorption uptake at room temperature over a period of 24 h for different hemp shiv samples with multiple coating layers.

It can be seen that hemp shiv absorbs a large amount of water within the first few minutes of dipping. Hemp shiv without any coatings shows the maximum water absorption with 400% increase in its original mass. This was mainly due to highly porous structure of hemp shiv as well as its tendency to absorb water due to its hydrophilic nature. A single coating layer on the hemp shiv reduces the water absorption by 200%. This proves the coating layer with hydrophobic functional groups provides water resistance to the hemp shiv. Further coating layers on the shiv show an increased water resistance which can be attributed to the decrease in porosity reported in the next section of this paper.

Based on our experimental data, a water absorption model was used to determine the parameters reported in Table 2. An empirical equation proposed by Tajvidi and Azad [38] described the dependence of water absorption on time as follows:

4 $${{\rm WA}}(t) = a\left( {1 - \exp \left( { - bt} \right)} \right)$$

where ${{\rm WA}}(t)$ is the water absorption (in percent), *a* and *b* are constants that are determined by the curve fitting procedure and *t* is the time in hours. This two parameter equation did not fit well with our experimental data.

**Table 2:** Water absorption parameters for hemp shiv samples

Coating layers	Eq. (5) parameters			
	*a*	*b*	*c*	*R* ^2^
Uncoated	1198.0	0.97 × 10^−7^	0.1327	0.9504
1	182.2	0.0033	0.334	0.9814
2	173.9	0.0006	0.2446	0.9975
5	93.89	0.0019	0.307	0.9821
8	72.81	0.0027	0.2978	0.9948
10	53.41	0.0066	0.2611	0.9365

Modifying Eq. (4) by an additional parameter [39, 40] describes our experimental data more accurately:

5 $${{\rm WA}}(t) = a(1 - \exp \left( { - bt} \right))^c$$

where *c* is the third constant determined by the curve fitting procedure

The values of WA and the constants (*a*, *b* and *c*) for uncoated and coated hemp shiv samples were obtained from curve fitting using MATLAB. Figure 6 shows that Eq. (5) fitted well with the water absorption data of the samples with *R*^2^ values varying between 0.9365 and 0.9975.

In our case, the coated hemp shiv samples reached equilibrium during the measurement which corresponds well with the saturation value given by parameter ‘*a*’ of Eq. (5). However, from the experimental data in Fig. 6, it can be seen that uncoated hemp shiv does not reach equilibrium and would continue to absorb water after the test. The curve fitting data for uncoated hemp shiv estimated that at saturation, the maximum water uptake would be 1200% increase in its initial mass. From Table 2, it can be seen that a single layer of sol–gel coating significantly reduces the parameter ‘*a*’ corresponding to the water absorption at saturation. This can be attributed to the water-repellent behaviour of the sol–gel coating on hemp shiv. Further coating layers enhance the water repellence of the hemp shiv thereby reducing their water uptake at saturation.

### Dynamic vapour sorption

**Fig. 7:**
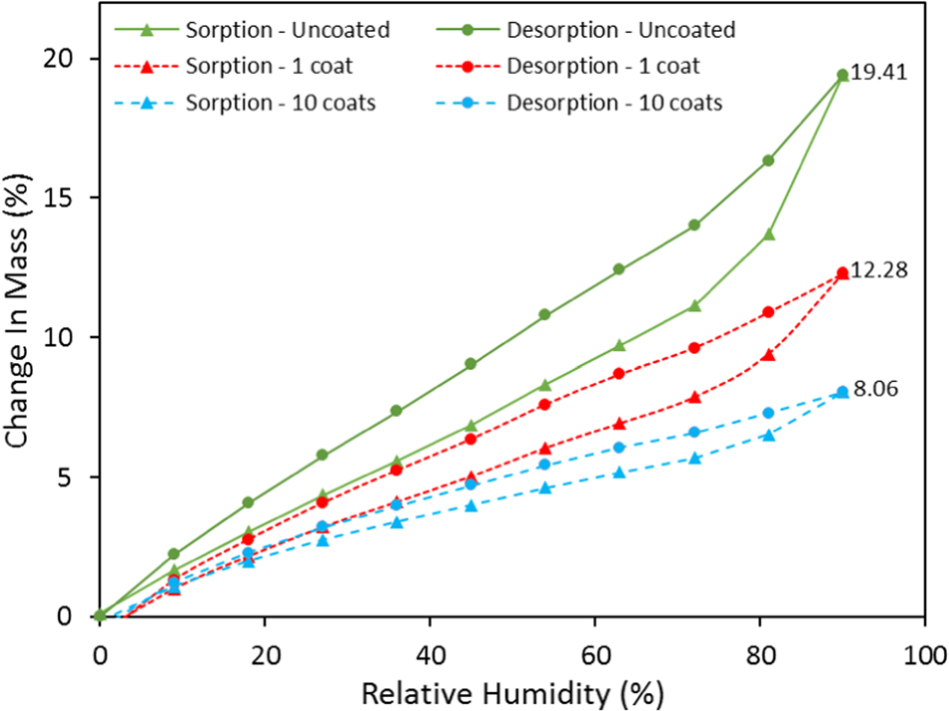
Adsorption–desorption isotherm by mass of uncoated and coated hemp shiv

The adsorption–desorption isotherm of uncoated and coated hemp shiv was determined at 23 °C using the DVS equipment over a RH range 0–90% (Fig. 7). The sol–gel coating caused a reduction in measured moisture content (MC) during the adsorption–desorption process. However, the reduced moisture content (MC_R_) of coated shiv calculated using Eq. (2) show only a marginal difference compared to the MC of uncoated shiv. This can be explained due to the fact that the mass of coated shiv (*m*_1_) is always higher than the mass of untreated hemp shiv (*m*_0_) due to coating layer. Therefore from Eqs. (1) and (2), MC = MC_R_ for uncoated hemp shiv but for coated shiv MC is lower than MC_R_ [33].

At the highest RH (90%), uncoated hemp shiv reached a MC of 19.41% whereas the values obtained for the MC of the coated shiv with 1 and 10 layers were 12.28% and 8.06%, respectively. Considering the mass increased due the sol–gel coating on the hemp shiv, the MC_R_ of the coated shiv with 1 and 10 layers were 16.21% and 17.34%, respectively.

**Fig. 8:**
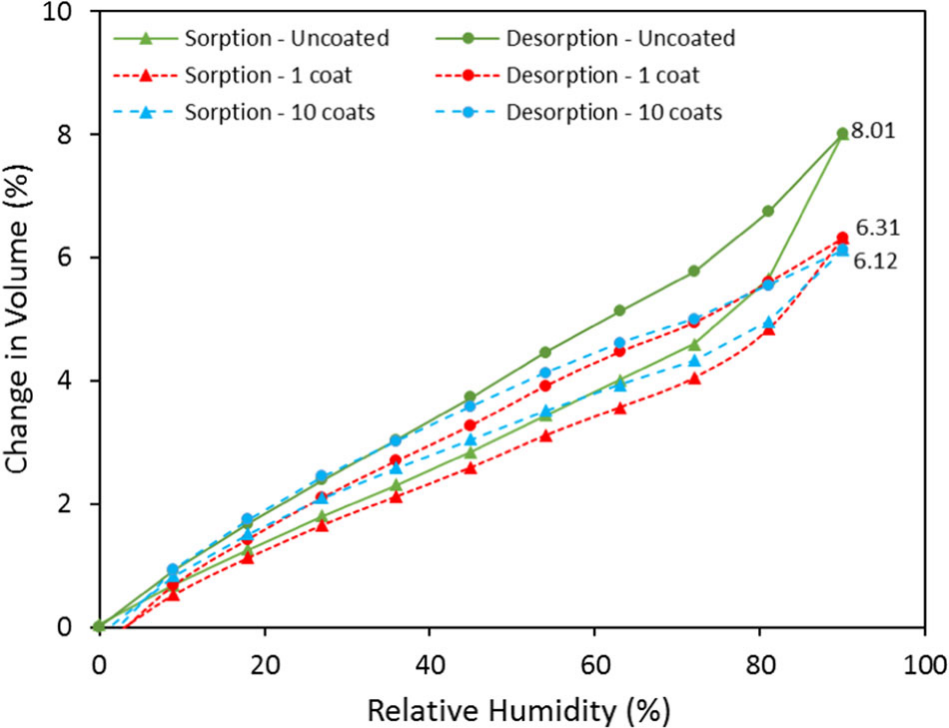
Adsorption–desorption isotherm by volume of uncoated and coated hemp shiv

Figure 8 represents the moisture adsorption data relating to volume of water condensed in the pores. From the DVS data, the mass of water adsorbed at each RH can be converted into volume and compared with the total accessible volume of hemp shiv. This analysis is valid based on the assumption that water is in the liquid state upon adsorption in the pores.

It can be seen from Figs. 7 and 8 that the curves for MC and MC_vol_ are different. The lower adsorption values in for the coated shiv in Fig. 7 are due to hydrophobic groups present on the surface of hemp shiv. However, the reduced hysteresis between the adsorption and desorption curves for the coated hemp shiv indicates that the condensed water does not penetrate deep into the shiv structure. The selected sol–gel formulation is able to provide only a certain level of hydrophobicity to the hemp shiv as the concentration of the MTES is kept constant for all the coating layers. The sol–gel coating interacts with the hydroxyl groups on hemp shiv thereby reducing mass of water adsorbed. Moreover, it can be seen in Fig. 8 that hemp shiv with 10 layers of sol–gel coating still adsorbs similar vol% of water as the single coated shiv due the presence of smaller pores that are not blocked by the sol–gel coating.

The high values of EMC can be explained by the fact that raw hemp shiv has a high content of cellulose with large number of accessible OH groups. However, modifying the hemp shiv surface with sol–gel coating blocks the free OH groups on the surface, thereby reducing the moisture adsorption capability to a certain extent but does not seal the pores.

### Porosity

**Fig. 9:**
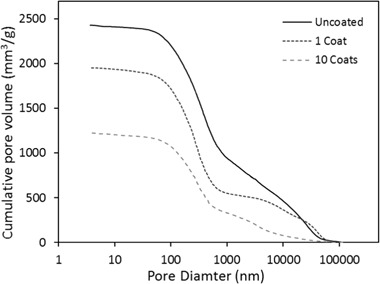
Pore volume distribution of uncoated and coated hemp shiv

**Fig. 10:**
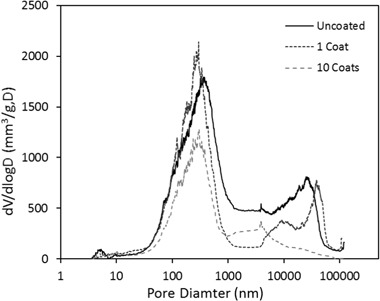
Pore size distribution of uncoated and coated hemp shiv

The porosity distribution of uncoated and coated shiv is given in Figs. 9 and 10. Raw hemp shiv shows higher porosity (78%) compared to the coated shiv with a single sol–gel layer (76%) and shiv with 10 layers of sol–gel coating (66%). From Fig. 9 it can be seen the uncoated hemp shiv has a larger cumulative pore volume compared to the coated shiv. This decrease in pore volume can be explained due the effect of the sol–gel coating reducing the size of the pores.

Figure 10 shows that the single sol–gel layer reduced the diameter of the larger pores, mainly in the range of 0.5 to 50 µm. Increasing the numbers of sol–gel layers on the shiv significantly reduce the pore size, possibly blocking some of the capillary pores completely. However, it may be noted that despite the single sol–gel layer on the shiv, the volume of smaller pores has increased and a refinement of the pore size in the range of 10 µm can be observed. It can be inferred that the deposition of the sol–gel coating has successfully taken place onto the hemp shiv surface. Therefore, the coated hemp shiv is capable of adsorbing moisture through the smaller pores whereas the water uptake is considerably reduced due to decrease in the larger pores as seen in the previous sections.

### Thermal analysis

**Fig. 11:**
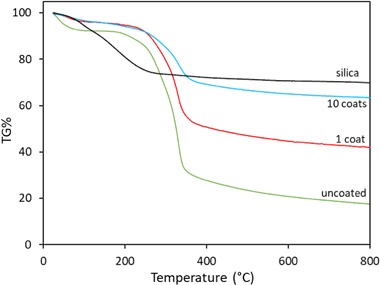
TGA thermograms of silica, uncoated and coated shiv samples

The TGA weight loss curves in a nitrogen atmosphere for silica, uncoated and sol–gel coated hemp shiv samples are shown in Fig. 11. The thermogravimetric profiles are summarised in Table 3. The uncoated and coated shiv samples degraded in three stages. The first stage which occurs below 100 °C mainly due to moisture evaporation, was higher in uncoated shiv compared to sol–gel coated shiv samples. This can be related to the binding of the silica network to the free hydroxyl groups in the coated shiv samples.

**Table 3:** Decomposition temperatures of silica, uncoated and coated hemp shiv

Samples	*T*_5_ (°C)	*T*_50_ (°C)	*T*_max_ (°C)	Residue
Uncoated shiv	50	325	329	17.5%
Shiv—1 coat	185	415	325	41.9%
Shiv—10 coats	180	–	334	63.5%
Silica	96	–	170	69.7%

The second degradation stage occurs around 300 °C which is due to the thermal depolymerisation of hemicellulose. The third peak observed at 330 °C is mainly due to cellulose and lignin decomposition. It can be seen from Fig. 10 that increasing the number of layers of the sol–gel coating on the shiv caused a shift in the TGA curves improving the thermal stability compared to the uncoated hemp shiv.

## Conclusion

Sol–gel technology has proved successful in modifying a highly hydrophilic bio-based material into a water-resistant building material. Deposited silica based sol–gel coating alters the morphology of hemp shiv, by penetrating the shiv structure thereby reducing the pore size and total pore volume of the hemp shiv. On one hand a cross linked network is formed between the silica (sol–gel coating) and the free hydroxyl groups on the surface of the shiv. On the other hand, the moisture sorption ability is not compromised which means the shiv retains its hygroscopic properties. Sol–gel coated hemp shiv showed improved thermal stability and good water resistance. It has also potential to be mixed with binders to produce composites with better interfacial adhesion, lower drying times, provide ease of handling during the manufacturing stage and ultimately a more robust bio-based thermal insulation building material.
